# Modeling the Geographic Spread of Rabies in China

**DOI:** 10.1371/journal.pntd.0003772

**Published:** 2015-05-28

**Authors:** Jing Chen, Lan Zou, Zhen Jin, Shigui Ruan

**Affiliations:** 1 Department of Mathematics, University of Miami, Coral Gables, Florida, United States of America; 2 Department of Mathematics, Sichuan University, Chengdu, Sichuan, People's Republic of China; 3 Complex Systems Research Center, Shanxi University Taiyuan, Shanxi, People's Republic of China; The Global Alliance for Rabies Control, UNITED STATES

## Abstract

In order to investigate how the movement of dogs affects the geographically inter-provincial spread of rabies in Mainland China, we propose a multi-patch model to describe the transmission dynamics of rabies between dogs and humans, in which each province is regarded as a patch. In each patch the submodel consists of susceptible, exposed, infectious, and vaccinated subpopulations of both dogs and humans and describes the spread of rabies among dogs and from infectious dogs to humans. The existence of the disease-free equilibrium is discussed, the basic reproduction number is calculated, and the effect of moving rates of dogs between patches on the basic reproduction number is studied. To investigate the rabies virus clades lineages, the two-patch submodel is used to simulate the human rabies data from Guizhou and Guangxi, Hebei and Fujian, and Sichuan and Shaanxi, respectively. It is found that the basic reproduction number of the two-patch model could be larger than one even if the isolated basic reproduction number of each patch is less than one. This indicates that the immigration of dogs may make the disease endemic even if the disease dies out in each isolated patch when there is no immigration. In order to reduce and prevent geographical spread of rabies in China, our results suggest that the management of dog markets and trades needs to be regulated, and transportation of dogs has to be better monitored and under constant surveillance.

## Author Summary

In 1999, human rabies cases were reported in about 120 counties in Mainland China, mainly in the southern provinces. Now outbreaks of human rabies have been reported in about 1000 counties and the disease has spread geographically from the south to the north. Phylogeographic analyses of rabies virus strains indicate that prevalent strains in northern provinces are indeed related to the remote southern provinces. It is believed that the geographical spread of rabies virus is caused by the transportation of dogs. In this paper, a multi-patch model is proposed to describe the spatial transmission dynamics of rabies in China and to investigate how the immigration of dogs affects the geographical spread of rabies. The expression and sensitivity analysis of the basic reproduction number indicates that the movement of dogs plays an essential role in the spatial transmission dynamics of rabies. Numerical simulations on the effect of the immigration rate in three pairs of provinces, Guizhou and Guangxi, Hebei and Fujian, Sichuan and Shaanxi, are also performed. It is shown that the immigration of dogs is the main factor for the long-distance inter-provincial spread of rabies and it is necessary to manage such inter-provincial transportation of dogs.

## Introduction

Rabies, as an acute and fatal zoonotic disease, is most often transmitted through the bite or scratch of a rabid animal. The rabies virus infects the central nervous system, ultimately causing disease in the brain and death. Once the symptoms of rabies have developed, its mortality rate is almost 100%. Rabies causes tens of thousands of deaths worldwide per year ([1]), more than 95% of which occur in Asia and Africa. More human deaths from rabies occur in Asia than anywhere else in the world ([2]). It was first recorded in ancient China in about 556 BC ([3]) and nowadays it is still a very serious public-health problem in China. It has been classified as a class II infectious disease in the National Stationary Notifiable Communicable Diseases and the annual data of human rabies have been archived by the Chinese Center for Disease Control and Prevention since 1950. From 1950 to 2013, 128,769 human rabies cases were reported in China ([4–7]), an average of 2,012 cases per year. It is estimated that 85%–95% of human rabies cases are due to dog bites in mainland China ([5]).

Recently, there are some studies on modeling the transmission dynamics of rabies in mainland China. Zhang et al. [8] proposed a deterministic model to study the transmission dynamics of rabies in China. The model consists of susceptible, exposed, infectious, and vaccinated subpopulations of both dogs and humans and describes the spread of rabies among dogs and from infectious dogs to humans. The model simulations agree with the human rabies data reported by the Chinese Ministry of Health from 1996 to 2010. It was shown that reducing dog birth rate and increasing dog immunization coverage rate are the most effective methods for controlling rabies in China and large scale culling of susceptible dogs can be replaced by immunization of them. Based on the model of Zhang et al. [8], Hou et al. [9] considered a deterministic model for the dog-human transmission of rabies, taking into account both domestic and stray dogs, and used the model to simulate the reported human cases in Guangdong Province, China. It was shown that the quantity of stray dogs also plays an important role in the transmission of rabies. Based on the fact that the monthly rabies data in China exhibit periodic patterns, Zhang et al. [10] constructed a susceptible, exposed, infectious, and vaccinated (SEIVS) model with periodic transmission rates to investigate the seasonal rabies epidemics. They evaluated the basic reproduction number, analyzed the dynamical behavior of the model, used the model to simulate the monthly data of human rabies cases reported by the Chinese Ministry of Health from January 2004 to December 2010, and explored some effective control measures for the rabies epidemics in China.

**Fig 1:**
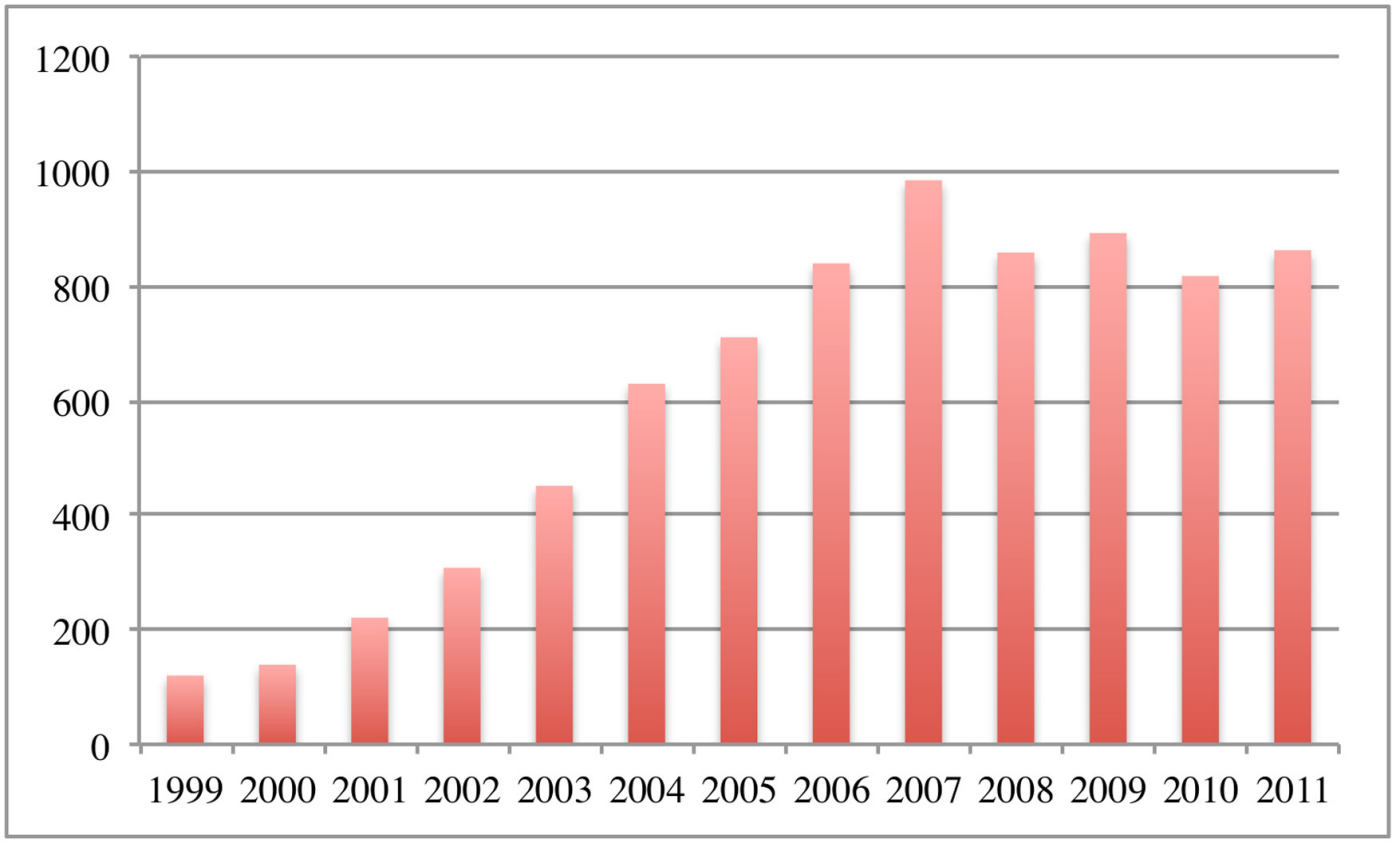
The number of counties in China with human rabies reported from 1999 to 2011.

In the last 20 years or so, rural communities and areas in Mainland China are invaded by rabies gradually. The range of infected hosts has expanded and the number of counties with reported human rabies increased significantly (See Fig 1). Moreover, human rabies has been expanded geographically from the south provinces to the central and north provinces (see [10]). Some provinces such as Shaanxi and Shanxi in the north, used to be rabies free, have reported more and more rabies cases in the past few years ([11]). Since the trade and transportation of dogs are regarded as the main cause for the spatial spread of rabies, Zhang et al. [10] extended their early ODE model to a reaction-diffusion model to study how the movement of dogs impacts the spatial spread of rabies. Their analysis indicates that the movement of dogs leads to the traveling wave of dog and human rabies and has a large influence on the minimal wave speed.

**Fig 2:**
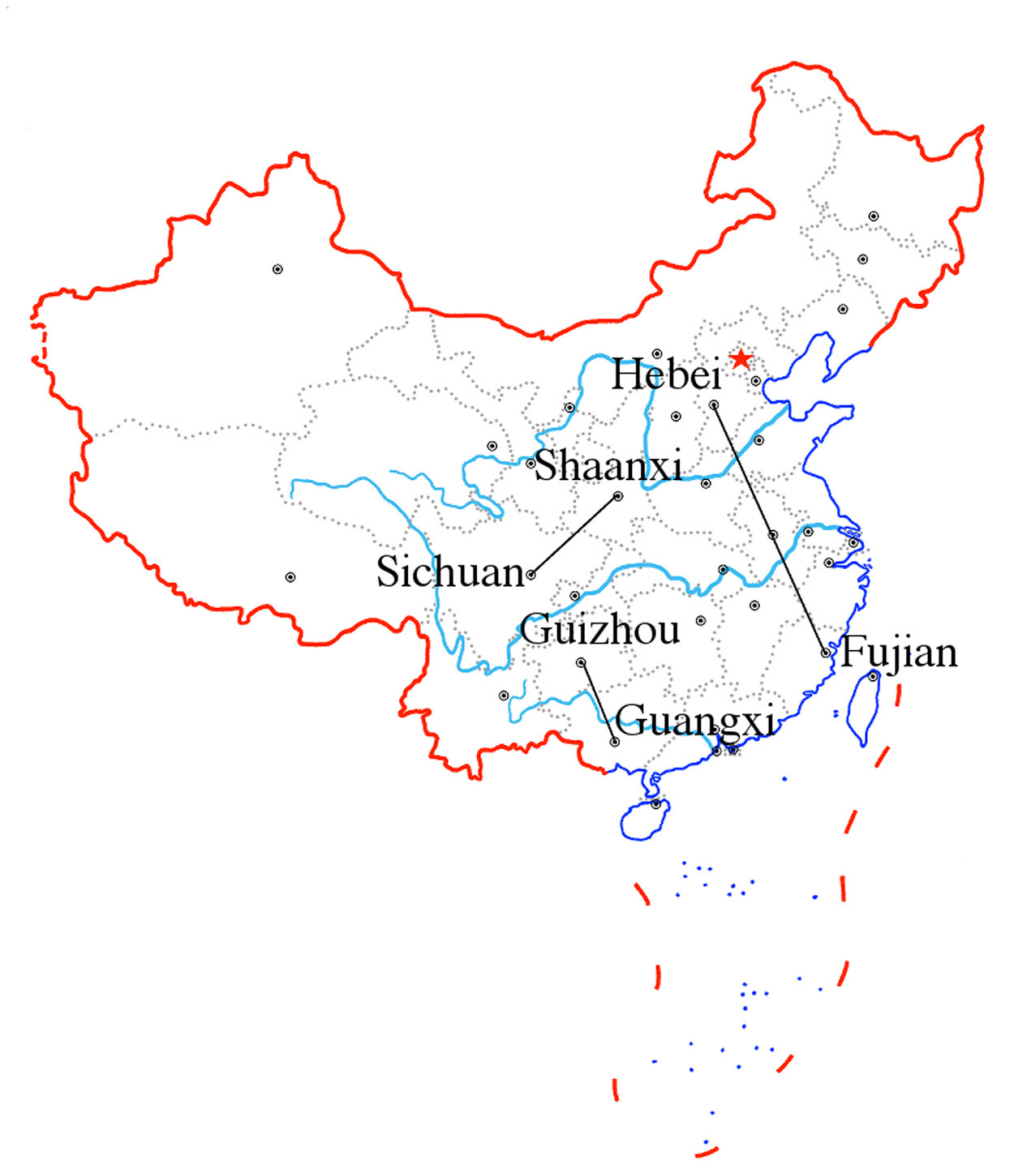
The linkage of rabies virus China I strain between Sichuan and Shaanxi, Guangxi and Guizhou, and Fujian and Hebei ([12, 14]).

Although dogs remain the major infection source, contributing 85%–95% of human cases in China ([5]), there are very little scientific studies and very few data on the population dynamics of dogs, let alone diseases of dogs. In order to improve rabies control and prevention, in 2005 the Chinese government implemented a trial surveillance program to monitor rabies at the national level in an attempt to obtain a more comprehensive epidemiological dataset. In addition to recording statistics on human cases, the Institute for Viral Disease Control and Prevention of China CDC cooperated with the provincial CDC laboratories and began collecting samples from dog populations in regions where human rabies cases had been reported. The positive samples were then submitted for DNA sequencing and combined with a second subset of selected sequences from publicly available sequences. Yu et al. [12] selected a subset of samples for sequencing and investigated the history and origin of the virus in China and examined the variation from a geographical perspective. Guo et al. [13] used comprehensive spatial analysis methodology to describe the spatiotemporal variation of human rabies infections in China from 2005 to 2011, detected spatiotemporal clusters of human rabies, modeled the transmission trend of rabies, and provided a scientific basis for improved targeted human rabies control interventions in China. Guo et al. [14] collected rabies virus nucleoprotein gene sequences from different provinces and investigated their phylogenetic and phylogeographic relationship. More specifically, their phylogeographical analyses of two rabies virus clades (China I and China II) lineages identified several provinces that appear to be epidemiologically linked and China I lineage plays the dominant role in the spread of rabies in China. Moreover, their analysis indicates that east China appears to be not only epidemiologically related to adjoining provinces but also to distant provinces, and seems to act as an epidemic hub for transmission of rabies virus to other regions, which is consistent with previous results by Yu et al. [12]. Other long distance translocations of rabies virus can also be identified as well as translocation events between neighboring provinces. Their analysis demonstrates a strong epidemiological linkage between Shaanxi to Sichuan and between Sichuan to Yunnan. This is consistent with surveillance data for human rabies cases which show dissemination of the virus from southwest China to neighboring provinces and into regions such as Shaanxi in the northern part of the county that have previously been incident free for several years (Yin et al. [11]). For both clades there appears to be a general trend of longitudinal transmission (Guangdong-Shandong, Fujian-Hebei, Zhejiang-Shandong) and latitudinal transmission (Yunnan-Shanghai, Guizhou-Shanghai, Hunan-Shanghai). That is also consistent with human rabies surveillance data which highlights a flow of cases from high incidence regions in the south of the country to medium and low incidence regions (Yin et al. [11]). For example, discrete phylogeographic analysis for China I strain ([12, 14]) indicates the linkage of rabies virus between Sichuan and Shaanxi, Guangxi and Guizhou, and Fujian and Hebei (Fig 2).

Zhang et al. [15] used a reaction-diffusion model to study the spatial spread of rabies in China. However, reaction-diffusion equations are based on the mathematical assumptions that the spatial domain is connected and the movement of dogs is a continuous process in the domain. While the phylogeographical analyses of rabies virus indicate that there are long distance inter-provincial spread of rabies in China, in order to investigate how the movement of dogs affects the geographic spread, we propose a multi-patch model to study the spatial transmission of rabies between dogs and from dogs to humans. We will describe the model in details, discuss the existence of the disease-free equilibrium, calculate the basic reproduction number, and study how the moving rates between patches affect the basic reproduction number. To investigate the epidemiological linkage (such as Guizhou and Guangxi, Hebei and Fujian, and Sichuan and Shaanxi) observed in Guo et al. [14], we will use the two-patch submodel to simulate the human rabies data to understand the inter-provincial spread of rabies in China.

## Methods

### Mathematical model

**Fig 3:**
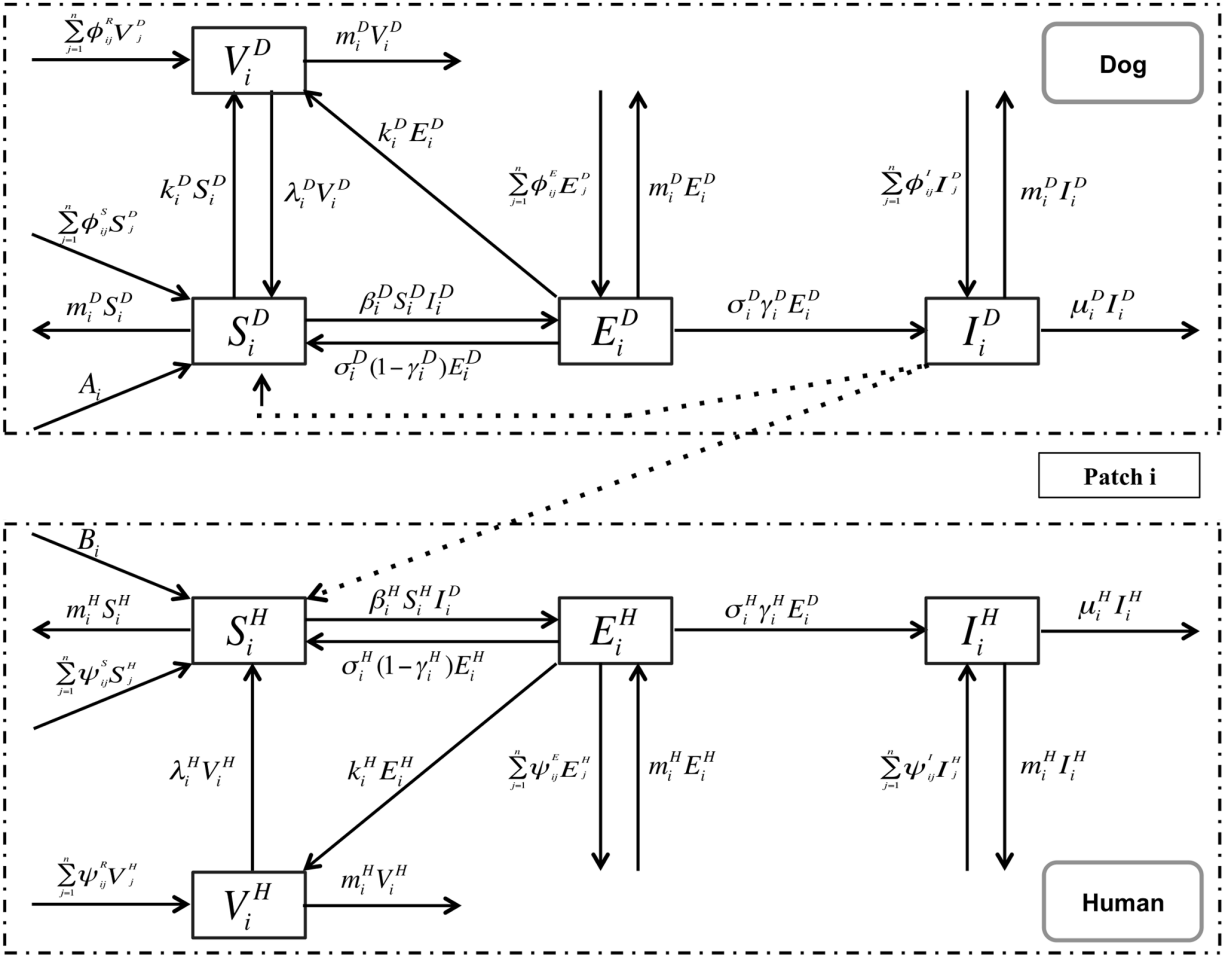
Flow chart for the transmission of rabies virus between dogs and from infectious dogs to humans within patches and from patches to patches via transportation of dogs.

Since the data on human rabies in mainland China are reported to the China CDC by provinces, we regard each provinces as a single patch and, in each patch, the submodel structure follows the SEIR model proposed by Zhang et al. [8] (see Fig 3). We use superscripts *H* and *D* to represent human and dog, respectively, and a subscript *i* to denote the *i*th-patch. We assume there are *n* patches where *n* ≥ 2 ([16]). For patch *i*, the dog population is divided into four subclasses: $S_{i}^{D}(t)$, $E_{i}^{D}(t)$, $I_{i}^{D}(t)$, and $V_{i}^{D}(t),$ which denote the populations of susceptible, exposed infectious and vaccinated dogs at time *t*, respectively. Similarly, the human population in patch *i* is classified into $S_{i}^{H}(t)$, $E_{i}^{H}(t)$, $I_{i}^{H}(t)$, and $V_{i}^{H}(t)$, which denote the populations of susceptible, exposed, infectious and vaccinated humans at time *t*, respectively. Our assumptions on the dynamical transmission of rabies between dogs and from dogs to humans are presented in the flowchart (Fig 3). The model in patch *i* is described by the following differential equations:

$$\begin{array}{c} \begin{array}{c} \frac{dS_{i}^{D}}{dt}=A_{i}+\lambda_{i}^{D}V_{i}^{D}+\sigma_{i}^{D}\left(1-\gamma_{i}^{D}\right)E_{i}^{D}-\beta_{i}^{D}S_{i}^{D}I_{i}^{D}-\left(m_{i}^{D}+k_{i}^{D}\right)S_{i}^{D}+\sum_{j=1}^{n}\phi_{ij}^{S}S_{j}^{D},\\ \frac{dE_{i}^{D}}{dt}=\beta_{i}^{D}S_{i}^{D}I_{i}^{D}-\left(m_{i}^{D}+\sigma_{i}^{D}+k_{i}^{D}\right)E_{i}^{D}+\sum_{j=1}^{n}\phi_{ij}^{E}E_{j}^{D},\\ \frac{dI_{i}^{D}}{dt}=\sigma_{i}^{D}\gamma_{i}^{D}E_{i}^{D}-\left(m_{i}^{D}+\mu_{i}^{D}\right)I_{i}^{D}+\sum_{j=1}^{n}\phi_{ij}^{I}I_{j}^{D},\\ \frac{dV_{i}^{D}}{dt}=k_{i}^{D}\left(S_{i}^{D}+E_{i}^{D}\right)-\left(m_{i}^{D}+\lambda_{i}^{D}\right)V_{i}^{D}+\sum_{j=1}^{n}\phi_{ij}^{V}V_{j}^{D},\\ \frac{dS_{i}^{H}}{dt}=B_{i}+\lambda_{i}^{H}V_{i}^{H}+\sigma_{i}^{H}\left(1-\gamma_{i}^{H}\right)E_{i}^{H}-m_{i}^{H}S_{i}^{H}-\beta_{i}^{H}S_{i}^{H}I_{i}^{D}+\sum_{j=1}^{n}\psi_{ij}^{S}S_{j}^{H},\\ \frac{dE_{i}^{H}}{dt}=\beta_{i}^{H}S_{i}^{H}I_{i}^{D}-\left(m_{i}^{H}+\sigma_{i}^{H}+k_{i}^{H}\right)E_{i}^{H}+\sum_{j=1}^{n}\psi_{ij}^{E}E_{j}^{H},\\ \frac{dI_{i}^{H}}{dt}=\sigma_{i}^{H}\gamma_{i}^{H}E_{i}^{H}-\left(m_{i}^{H}+\mu_{i}^{H}\right)I_{i}^{H}+\sum_{j=1}^{n}\psi_{ij}^{I}I_{j}^{H},\\ \frac{dV_{i}^{H}}{dt}=k_{i}^{H}E_{i}^{H}-\left(m_{i}^{H}+\lambda_{i}^{H}\right)V_{i}^{H}+\sum_{j=1}^{n}\psi_{ij}^{V}V_{j}^{H}. \end{array} \end{array}$$ (1)

All parameters and their interpretations are listed in Table 1.

**Table 1:** Parameters and descriptions.

Parameter	Description	Reference
*A*_*i*_	the annual birth rate of dogs in patch *i*	estimation
$\lambda_{i}^{D}$	the loss rate of vaccination immunity for dogs in patch *i*	[19]
$\frac{1}{\sigma_{i}^{D}}$	the time duration in which infected dogs in patch *i* remain infectious	[18]
$\gamma_{i}^{D}$	the risk factor of clinical outcome of exposed dogs in patch *i*	[5]
$m_{i}^{D}$	the non-disease related death rate for dogs in patch *i*	[24]
$k_{i}^{D}$	the vaccination rate of dogs in patch *i*	[5]
$\mu_{i}^{D}$	the disease-related death rate for dogs in patch *i*	[5]
$\beta_{i}^{D}$	the transmission coefficient of infectious dogs to susceptible dogs in patch *i*	fitting
*B*_*i*_	the annual birth rate of humans in patch *i*	[17]
$\lambda_{i}^{H}$	the loss rate of vaccination immunity of humans in patch *i*	[25]
$\frac{1}{\sigma_{i}^{H}}$	the time duration of infectiousness of infected humans in patch *i*	[2]
$\gamma_{i}^{H}$	the risk factor of clinical outcome of exposed humans in patch *i*	[20]
$m_{i}^{H}$	the natural death rate of humans in patch *i*	[17]
$k_{i}^{H}$	the vaccination rate of humans in patch *i*	[5]
$\mu_{i}^{H}$	the disease-related death rate of humans in patch *i*	[5]
$\beta_{i}^{H}$	the transmission coefficient of infectious dogs to susceptible humans in patch *i*	fitting
$\phi_{ij}^{K}\geq 0$ (*K* = *S*, *E*, *I*, *V*)	the immigration rate from patch *j* to patch *i* for *i* ≠ *j* of susceptible (exposed, infectious, and vaccinated) dogs	fitting
$\psi_{ij}^{K}\geq 0$ (*K* = *S*, *E*, *I*, *V*)	the immigration rate from patch *j* to patch *i* for *i* ≠ *j* of susceptible (exposed, infectious, and vaccinated) humans	fitting

*A*_*i*_ describes the annual birth rate of the dog population in patch *i*; $\beta_{i}^{D}$ denotes the transmission coefficient between dogs in patch *i* and $\beta_{i}^{D}S_{i}^{D}I_{i}^{D}$ describes the transmission of rabies from infectious dogs to susceptible dogs in this patch; $1/\sigma_{i}^{D}$ represents the incubation period of infected dogs in patch *i*; $\gamma_{i}^{D}$ is the risk factor of clinical outcome of exposed dogs in patch *i*. Therefore, $\sigma_{i}^{D}\gamma_{i}^{D}E_{i}^{D}$ denotes dogs that develop clinical rabies and enter the susceptible class and the rest $\sigma_{i}^{D}(1-\gamma_{i}^{D})E_{i}^{D}$ denotes the exposed dogs that do not develop clinical rabies; $m_{i}^{D}$ is the non-disease related death rate for dogs in patch *i*; $k_{i}^{D}$ is the vaccination rate of dogs and $\lambda_{i}^{D}$ denotes the loss rate of vaccination immunity for dogs in patch *i*; $\mu_{i}^{D}$ is the disease-related death rate for dogs in patch *i*.

For the human population, similarly *B*_*i*_ describes the annual birth rate of the human population in patch *i*; $\beta_{i}^{H}$ denotes the transmission coefficient from dogs to humans in patch *i* and $\beta_{i}^{H}S_{i}^{H}I_{i}^{D}$ describes the transmission of rabies from infectious dogs to susceptible dogs in this patch; $1/\sigma_{i}^{H}$ represents the incubation period of infected humans in patch *i*; $\sigma_{i}^{H}\gamma_{i}^{H}E_{i}^{H}$ describes exposed people that become infectious and $\sigma_{i}^{H}(1-\gamma_{i}^{H})E_{i}^{H}$ describes the exposed people that return to be susceptible; $m_{i}^{H}$ is the non-disease related death rate for humans in patch *i*; $k_{i}^{H}$ is the vaccination rate of dogs and $\lambda_{i}^{H}$ denotes the loss rate of vaccination immunity for huamns in patch *i*; $\mu_{i}^{H}$ is the disease-related death rate for humans in patch *i*.

$\phi_{ij}^{K}\geq 0$ (*K* = *S*, *E*, *I*, *V*) is the immigration rate from patch *j* to patch *i* for *i* ≠ *j* of susceptible, exposed, infectious, and vaccinated dogs, respectively; $\psi_{ij}^{K}\geq 0$ (*K* = *S*, *E*, *I*, *V*) is the immigration rate from patch *j* to patch *i* for *i* ≠ *j* of susceptible, exposed, infectious, and vaccinated humans, respectively. Then $\sum_{j\neq i}\phi_{ij}^{K}K_{i}^{D}$ (*K* = *S*, *E*, *I*, *V*) describes the corresponding subclass of the dog population that enter into patch *i* from other patches and $\sum_{j\neq i}\phi_{ji}^{K}K_{i}^{D}$ denotes the corresponding subclass dog population that leave patch *i*. Meanwhile, the immigrations of humans are described in the same way by $\psi_{ij}^{K}$ (K = S, E, I, V).

### Data and parameters

Data used to simulate our model are from the Data-Center of China Public Health Science reported by China CDC. After the 2003 SARS outbreak, the Chinese government strengthened its public health disease surveillance system. From 2004, the digital monthly reporting system has been replaced by a web-based, real-time reporting system which covers 39 diseases across all regions of the country. Each case is reported with the detailed information including sex, age, date of infection, diagnosis and death, the address of reporting hospital, and the reporting district administrative code. This well-established surveillance system provides valuable data for mathematical modelers in studying these infectious diseases.

We used a two-patch submodel to simulate the data of human rabies from 2004 to 2012 in three pairs of provinces: Guangxi and Guizhou, Fujian and Hebei, and Sichuan and Shaanxi (see Fig 2). Each province is regarded as a patch in the model (*n* = 2). The parameters about humans inculding the annual birth rate and natural death rate of humans in each province are adopted from the “China Health Statistical Yearbook 2012” ([17]). The incubation period for rabies is typically 1–3 months ([2]), we assume that it is 2 months on average, thus $\sigma_{i}^{H}=6/year$. Similarly, we also have $\sigma_{i}^{H}=10/year$ ([18]). The disease induced death rates of humans and dogs are assumed to be 1 ([5]). According to [5], the vaccination rate $k_{i}^{H}$ of humans in China is about 0.5 and the risk factor of clinical outcome of exposed dogs $\gamma_{i}^{D}$ is 0.4. Based on studies the minimum duration of immunity for canine is 3 years ([19]), we assume that the loss rate of vaccination immunity for dogs in patch *i* is $\lambda_{i}^{D}=1/3/yaer\approx 0.33/year$. Rabies mortality after untreated bites by rabid dogs varies from 38% to 57% ([20]), thus we take the average 47.5% as the risk factor of clinical outcome of exposed humans.

The difficulty in parameter estimations is that there is no scientifically or officially reported data on dogs in China. So the values of *A*_*i*_ used in simulations are estimated based on the dog density from the household survey ([21]), the total areas of provinces, the density of human population and other research results ([9, 10, 15]). Now we assume that the immigration rates of susceptible, exposed, infectious and vaccinated dogs are same. Additionally, susceptible, exposed and vaccinated humans also move in the same rate but infectious humans do not move inter-provincially which is set as $\psi_{ij}^{H}=0$. All other parameters are left to be unknown and estimated through simulating the model by the data.

### Basic reproduction number and sensitivity analysis

The basic reproduction number ℛ_0_ is defined as the expected number of secondary cases produced by a typical infection in a completely susceptible population ([22]). Here, the basic reproduction number of rabies which reflects the expected number of dogs infected by a single infected dog, is derived from the mathematical model that describes the transmission dynamics of rabies following the method in van den Driessche and Watmough [23]. Mathematically, *R*_0_ is defined as the dominant eigenvalue of a linear operator. In S1 Text, the overall basic reproduction number ℛ_0_ for the whole system is calculated. The isolated basic reproduction number,

$$\begin{array}{c} {𝓡}_{0}^{i}=\frac{\beta_{i}^{D}\sigma_{i}^{D}\gamma_{i}^{D}A_{i}\left(m_{i}^{D}+\lambda_{i}^{D}\right)}{m_{i}^{D}\left(m_{i}^{D}+\mu_{i}^{D}\right)\left(m_{i}^{D}+\sigma_{i}^{D}+k_{i}^{D}\right)\left(m_{i}^{D}+\lambda_{i}^{D}+k_{i}^{D}\right)}, \end{array}$$ (2)

is the basic reproduction number in one single patch (patch *i* here) when all the immigration rates are zero. That is the basic reproduction number in an isolated patch under the assumption that there is no immigration at all. For the two-patch submodel, *R*_0_ can be expressed as

$$\begin{array}{cc} R_{0}= & \left(\beta_{1}^{D}S_{1}^{D*}\sigma_{1}^{D}\gamma_{1}^{D}(m_{2}^{D}+\sigma_{2}^{D}+k_{2}^{D}+\phi_{12}^{E})(m_{2}^{D}+\mu_{2}^{D}+\phi_{12}^{I}\right)\\ & +\beta_{2}^{D*}S_{2}^{D*}\sigma_{2}^{D}\gamma_{2}^{D}(m_{1}^{D}+\sigma_{1}^{D}+k_{1}^{D}+k_{1}^{D}+\phi_{21}^{E})(m_{1}^{D}+\mu_{1}^{D}+\phi_{21}^{I})\\ & +\beta_{2}^{D}S_{2}^{D*}\sigma_{1}^{D}\gamma_{1}^{D}\phi_{12}^{E}\phi_{21}^{I}+\beta_{1}^{D}S_{1}^{D*}\sigma_{2}^{D}\gamma_{2}^{D}\phi_{21}^{E}\phi_{12}^{I}\\ & +((\beta_{1}^{D}S_{1}^{D*}\sigma_{1}^{D}\gamma_{1}^{D}(m_{2}^{D}+\sigma_{2}^{D}+k_{2}^{D}+\phi_{21}^{E})(m_{2}^{D}+\mu_{2}^{D}+\phi_{12}^{I})\\ & +\beta_{2}^{D}S_{2}^{D*}\sigma_{2}^{D}\gamma_{2}^{D}(m_{1}^{D}+\sigma_{1}^{D}+k_{1}^{D}+\phi_{21}^{E})(m_{1}^{D}+\mu_{1}^{D}+\phi_{21}^{I})\\ & {+\phi_{12}^{E}\phi_{21}^{I}\beta_{2}^{D}S_{2}^{D*}\sigma_{1}^{D}\gamma_{1}^{D}+\phi_{21}^{E}\phi_{12}^{I}\beta_{1}^{D}S_{1}^{D*}\sigma_{2}^{D}\gamma_{2}^{D})}^{2}\\ & -4((m_{1}^{D}+\sigma_{1}^{D}+k_{1}^{D}+\phi_{21}^{E})(m_{2}^{D}+\sigma_{2}^{D}+k_{2}^{D}+\phi_{12}^{E})-\phi_{21}^{E}\phi_{12}^{E})\\ & {\left((m_{1}^{D}+\mu_{1}^{D}+\phi_{21}^{I})(m_{2}^{D}+\mu_{2}^{D}+\phi_{12}^{I})-\phi_{21}^{I}\phi_{12}^{I}(\beta_{1}^{D}S_{1}^{D*}\beta_{2}^{D}S_{2}^{D*}\sigma_{1}^{D}\gamma_{1}^{D}\sigma_{2}^{D}\gamma_{2}^{D})\right)}^{\frac{1}{2}}/\\ & \left\{2((m_{1}^{D}+\sigma_{1}^{D}+k_{1}^{D}+\phi_{21}^{E})(m_{2}^{D}+\sigma_{2}^{D}+k_{2}^{D}+\phi_{12}^{E})-\phi_{21}^{E}\phi_{12}^{E}\right)\\ & ((m_{1}^{D}+\mu_{1}^{D}+\phi_{21}^{I})(m_{2}^{D}+\mu_{2}^{D}+\phi_{21}^{I})(m_{2}^{D}+\mu_{2}^{D}+\phi_{12}^{I})-\phi_{21}^{I}\phi_{12}^{I}\}. \end{array}$$ (3)

The value of *R*_0_ gives an important threshold that determines if the disease will die out or not eventually. Roughly speaking, if ℛ_0_ > 1 each primary infected dog averagely will produce more than one secondary infected dog. Therefore the disease will persist. Conversely, if ℛ_0_ < 1 the expected number of secondary case produces by the primary case is less than one. Thus the disease will die out. The purpose is to reduce *R*_0_ by possible disease control strategies. However, the formula is very complicated and impossible to analyze the relationship between the parameters and ℛ_0_ even for a two-patch model. Sensitivity analysis can aid in discovering how each parameter quantitatively affects *R*_0_. Furthermore, we will study how the immigration rate affect the basic reproduction numbers of the whole system and the isolated patchs by performing some sensitivity analyses.

## Results

In this section, we first use the two-patch submodel to simulate the reported human rabies data from Guangxi and Guizhou, Sichuan and Shaanxi, and Fujian and Hebei, respectively. Then we carry out some sensitivity analyses of the basic reproduction number in terms of some parameters of dogs, especially the immigration rates between provinces.

### Numerical simulations

**Fig 4:**
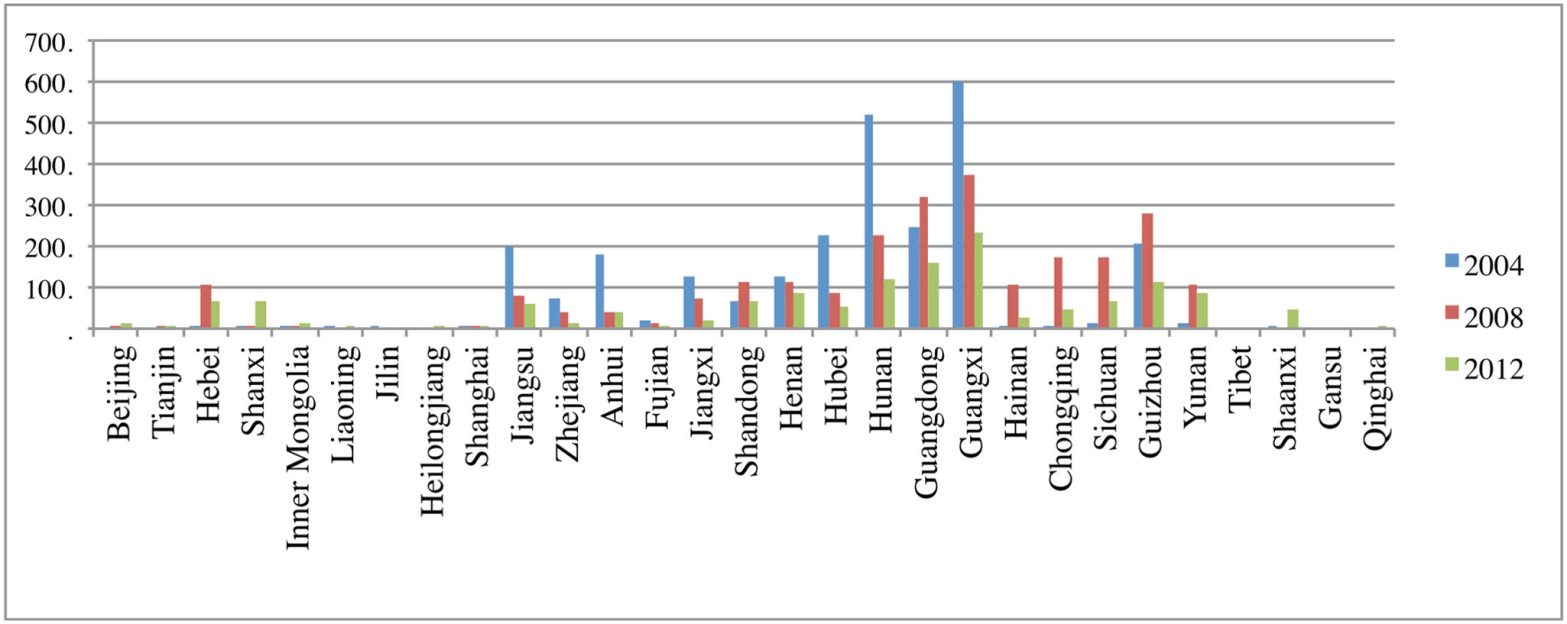
Reported human rabies cases by provinces in mainland China in 2004, 2008, and 2012 (data from [25]).

Fig 4 presents the reported human rabies cases in different provinces in Mainland China in the years 2004, 2008, and 2012. Although the numbers of cases decrease in some of the endemic provinces such as Guangxi and Hunan, some other provinces such as Shanxi and Shaanxi keep increasing. Some non-endemic provinces are becoming endemic in recent years. For example, Hebei, Shanxi and Shaanxi. Discrete phylogeographic analysis for China I strain ([12, 14]) indicates the linkage of rabies virus between Sichuan and Shaanxi, Guangxi, and Guizhou, and Fujian and Hebei (Fig 2).

**Fig 5:**
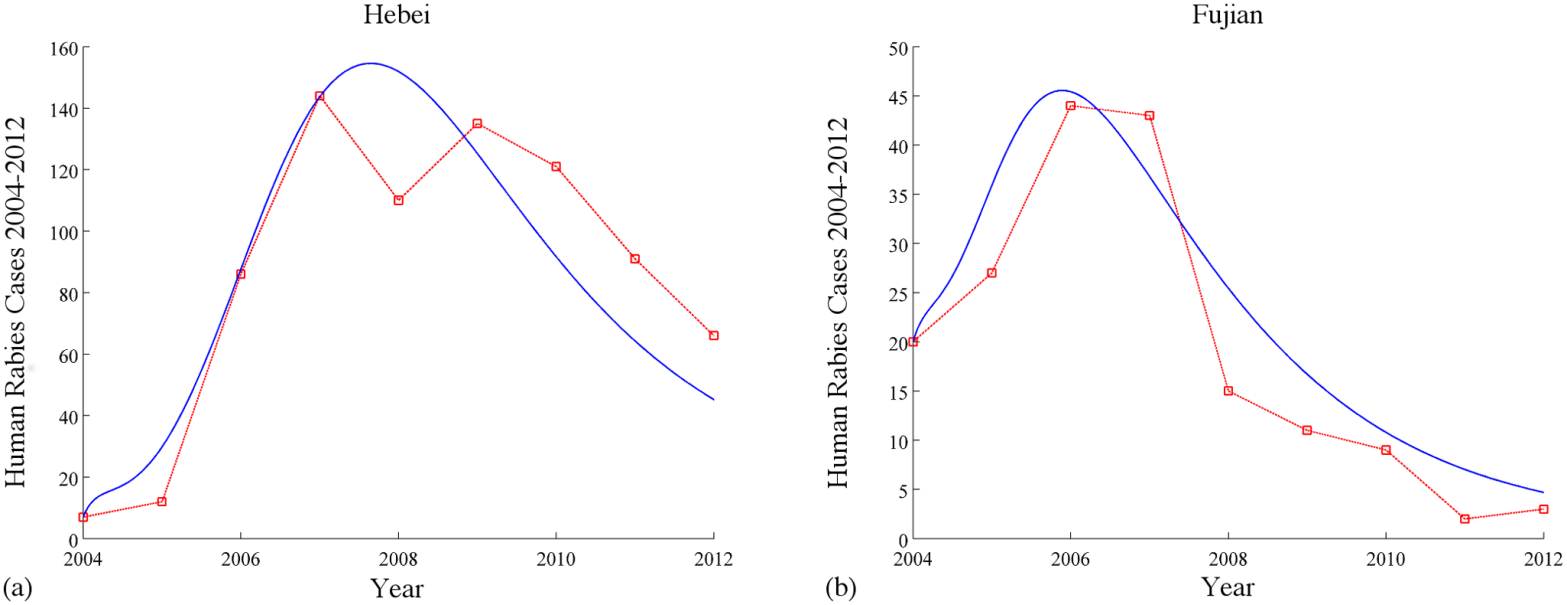
Simulations of the numbers of reported human rabies cases in Hebei and Fujian from 2004 to 2012. The solid blue curves are simulation results by our model and the dashed red curves are the numbers of reported cases by China CDC [25]. Values of parameters: *A*_1_ = 3 × 10^5^, *A*_2_ = 5 × 10^5^, *B*_1_ = 8.797 × 10^5^, *B*_2_ = 4.101 × 10^5^, $\lambda_{1}^{D}=\lambda_{2}^{D}=0.33$, $\lambda_{1}^{H}=\lambda_{2}^{H}=2$, $\sigma_{1}^{D}=\sigma_{2}^{D}=10$, $\sigma_{1}^{H}=\sigma_{2}^{H}=6$, $\gamma_{1}^{D}=\gamma_{2}^{D}=0.4$, $\gamma_{1}^{H}=\gamma_{2}^{H}=0.475$, $m_{1}^{D}=m_{2}^{D}=0.345$, $m_{1}^{H}=m_{2}^{H}=0.00662$, $k_{1}^{D}=0.09$, $k_{2}^{D}=0.00$, $k_{1}^{H}=k_{2}^{H}=0.5$, $\mu_{1}^{D}=\mu_{2}^{D}=\mu_{1}^{H}=\mu_{2}^{H}=1$, $\beta_{1}^{D}=2.45\times 10^{-6}$, $\beta_{2}^{D}=2.2\times 10^{-6}$, $\beta_{1}^{H}=2.2\times 10^{-11}$, $\beta_{2}^{H}=1.4\times 10^{-11}$, $\phi_{12}^{S}=\phi_{12}^{E}=\phi_{12}^{I}=\phi_{12}^{V}=0.6$, $\phi_{21}^{S}=\phi_{21}^{E}=\phi_{21}^{I}=\phi_{21}^{V}=0.05$, $\psi_{12}^{S}=\psi_{12}^{E}=\psi_{12}^{V}=0.2$, $\psi_{21}^{S}=\psi_{21}^{E}=\psi_{21}^{V}=0.32$, $\psi_{12}^{I}=\psi_{21}^{I}=0$.

*(a) Hebei and Fujian*. From Guo et al. [14], we know that Hebei and Fujian are epidemiologically linked. In Hebei, there was only one human rabies case reported in 2000 ([5]), while it is now one of the 15 provinces having more than 1,000 cumulative cases and is included in “Mid-to-long-term Animal Disease Eradication Plan for 2012–2020” project. We take Hebei and Fujian as two patches in model Eq (1) (when *n* = 2) and simulate the numbers of human cases from 2004 to 2012 by the model. In Fig 5, the solid blue curves represent simulation results and the dashed red curves are reported numbers of human rabies cases from 2004 to 2012, which show a reasonable match between the simulation results and reported data from China CDC. Based on the values of parameters in the simulations and the formula of the basic reproduction number in the two-patch model, we calculated that ℛ_0_ = 1.0319. That means the disease will not die out in this two-patch system.

Interestingly, now we assume there is no immigration of both dogs and humans in this system and calculated the isolated basic reproduction number in each province. The isolated basic reproduction numbers for Hebei and Fujian are ${ℛ}_{0}^{\text{Hebei}}=0.5477$ and ${ℛ}_{0}^{\text{Fujian}}=0.8197$, respectively. Under this assumption the disease would die out in both provinces since their isolated basic reproduction number is less than one. This example theoretically shows the possibility that the immigration of dogs can lead the disease to a worse scenario even it could be eliminated in each isolated patch. It is remarkable that we only mentioned the dog immigration here because a simple observation to the formula of the basic reproduction number in the S1 Text shows that only the immigration rates of dogs ($\phi_{ij}^{K}$ for *K* = *S*, *E*, *I*, *V*) can affect it. In fact, only dogs can carry the rabies virus and then spread it to humans and other dogs. This transmission feature supports our mathematical analysis.

**Fig 6:**
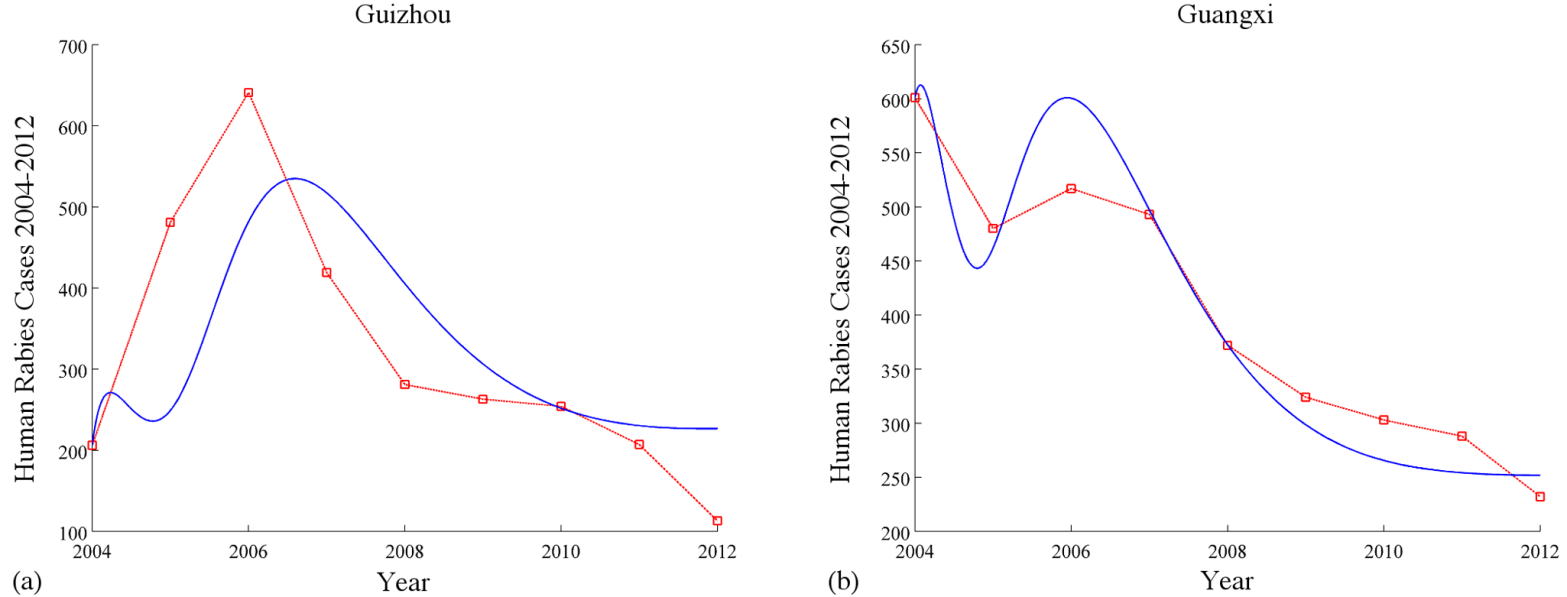
Simulations of the numbers of reported human rabies cases in Guizhou and Guangxi from 2004 to 2012. The solid blue curves are simulation results by our model and the dashed red curves are the numbers of reported cases by China CDC [25]. Values of parameters: *A*_1_ = 3 × 10^6^, *A*_2_ = 8.5 × 10^5^, *B*_1_ = 7.223 × 10^5^, *B*_2_ = 5.143 × 10^5^, $\lambda_{1}^{D}=\lambda_{2}^{D}=0.33$, $\lambda_{1}^{H}=\lambda_{2}^{H}=2$, $\sigma_{1}^{D}=\sigma_{2}^{D}=10$, $\sigma_{1}^{H}=\sigma_{2}^{H}=6$, $\gamma_{1}^{D}=\gamma_{2}^{D}=0.4$, $\gamma_{1}^{H}=\gamma_{2}^{H}=0.475$, $m_{1}^{D}=m_{2}^{D}=0.345$, $m_{1}^{H}=m_{2}^{H}=0.00662$, $k_{1}^{D}=0.07$, $k_{2}^{D}=0.05$, $k_{1}^{H}=k_{2}^{H}=0.5$, $\mu_{1}^{D}=\mu_{2}^{D}=\mu_{1}^{H}=\mu_{2}^{H}=1$, $\beta_{1}^{D}=2.7\times 10^{-6}$, $\beta_{2}^{D}=2.4\times 10^{-6}$, $\beta_{1}^{H}=1.15\times 10^{-11}$, $\beta_{2}^{H}=2.8\times 10^{-11}$, $\phi_{12}^{S}=\phi_{12}^{E}=\phi_{12}^{I}=\phi_{12}^{V}=0.2$, $\phi_{21}^{S}=\phi_{21}^{E}=\phi_{21}^{I}=\phi_{21}^{V}=0.15$, $\psi_{12}^{S}=\psi_{12}^{E}=\psi_{12}^{V}=0.15$, $\psi_{21}^{S}=\psi_{21}^{E}=\psi_{21}^{V}=0.2$, $\psi_{12}^{I}=\psi_{21}^{I}=0$.

*(b) Guizhou and Guangxi*. A statistically significant translocation event is also predicted between Guizhou and Guangxi in Yu et al. [12]. Fig 4 shows that Guizhou and Guangxi have large numbers of human rabies cases (both are in top 5 endemic provinces in China) in recent years. Particularly, the number of human deaths caused by rabies virus in Guangxi is ranked the highest in China. Similar simulations were carried out here to these two provinces and results are shown in Fig 6. The isolated basic reproduction numbers for Guizhou and Guangxi are calculated as ${ℛ}_{0}^{\text{Guizhou}}=1.5998$ and ${ℛ}_{0}^{\text{Guangxi}}=6.1905$, respectively, while the basic reproduction number for the whole system is estimated to be ℛ_0_ = 4.9211. To eliminate rabies we need some effective control strategies that can reduce ℛ_0_ significantly. Thus it is even more challenging to control and prevent the disease in Guangxi and Guizhou from a numerical perspective.

**Fig 7:**
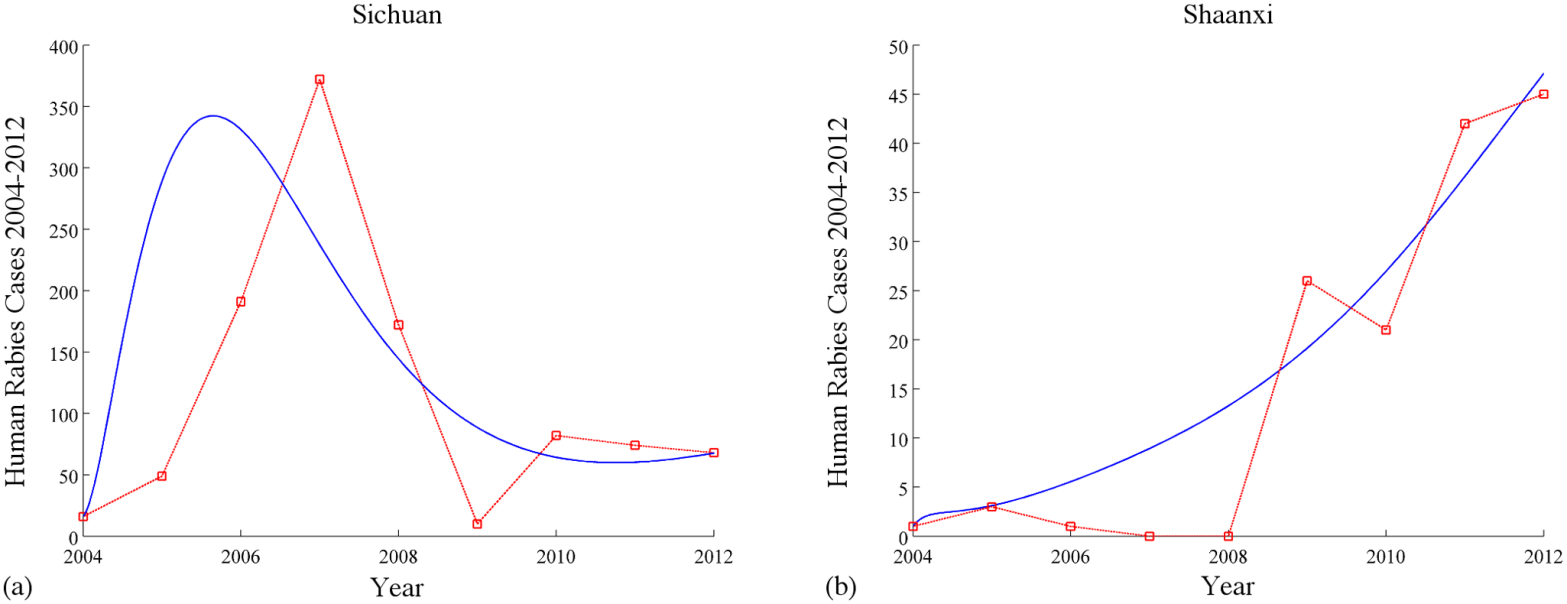
Simulations of the numbers of reported human rabies cases in Sichuan and Shaanxi from 2004 to 2012. The solid blue curves are simulation results by our model and the dashed red curves are the numbers of reported cases by China CDC [25]. Values of parameters: *A*_1_ = 3 × 10^5^, *A*_2_ = 3 × 10^5^, *B*_1_ = 7.184 × 10^5^, *B*_2_ = 3.634 × 10^5^, $\lambda_{1}^{D}=\lambda_{2}^{D}=0.33$, $\lambda_{1}^{H}=\lambda_{2}^{H}=2$,$\sigma_{1}^{D}=\sigma_{2}^{D}=10$, $\sigma_{1}^{H}=\sigma_{2}^{H}=6$, $\gamma_{1}^{D}=\gamma_{2}^{D}=0.4$,$\gamma_{1}^{H}=\gamma_{2}^{H}=0.475$, $m_{1}^{D}=m_{2}^{D}=0.345$, $m_{1}^{H}=m_{2}^{H}=0.00662$, $k_{1}^{D}=0.09$, $k_{2}^{D}=0.09$, $k_{1}^{H}=k_{2}^{H}=0.5$, $\mu_{1}^{D}=\mu_{2}^{D}=\mu_{1}^{H}=\mu_{2}^{H}=1$, $\beta_{1}^{D}=2.7\times 10^{-6}$, $\beta_{2}^{D}=2.4\times 10^{-6}$, $\beta_{1}^{H}=9.5\times 10^{-10}$, $\beta_{2}^{H}=4\times 10^{-11}$, $\phi_{12}^{S}=\phi_{12}^{E}=\phi_{12}^{I}=\phi_{12}^{V}=0.03$, $\phi_{21}^{S}=\phi_{21}^{E}=\phi_{21}^{I}=\phi_{21}^{V}=0.4$, $\psi_{12}^{S}=\psi_{12}^{E}=\psi_{12}^{V}=0.3$, $\psi_{21}^{S}=\psi_{21}^{E}=\psi_{21}^{V}=0.05$, $\psi_{12}^{I}=\psi_{21}^{I}=0.$

*(c) Sichuan and Shaanxi*. Shaanxi, which is now an alarming province for rabies in China, had only 15 cumulative human cases from 2000 to 2006 (only 2 to 3 cases every year on average). However, 26 human cases were reported in 2009 and the number keeps increasing after that. Rabies was found to spread along the road network [13]. With the parameters in Fig 7, the isolated basic reproduction numbers for Sichuan and Shaanxi are ${ℛ}_{0}^{\text{Sichuan}}=1.3414$ and ${ℛ}_{0}^{\text{Shaanxi}}=1.0061$, respectively, while the basic reproduction number for the two provinces with immigration is ℛ_0_ = 1.5085 which is greater than both of these two isolated ones. Numerically, that means more efforts may be needed to eliminate the virus in humans if the immigration is involved.

**Fig 8:**
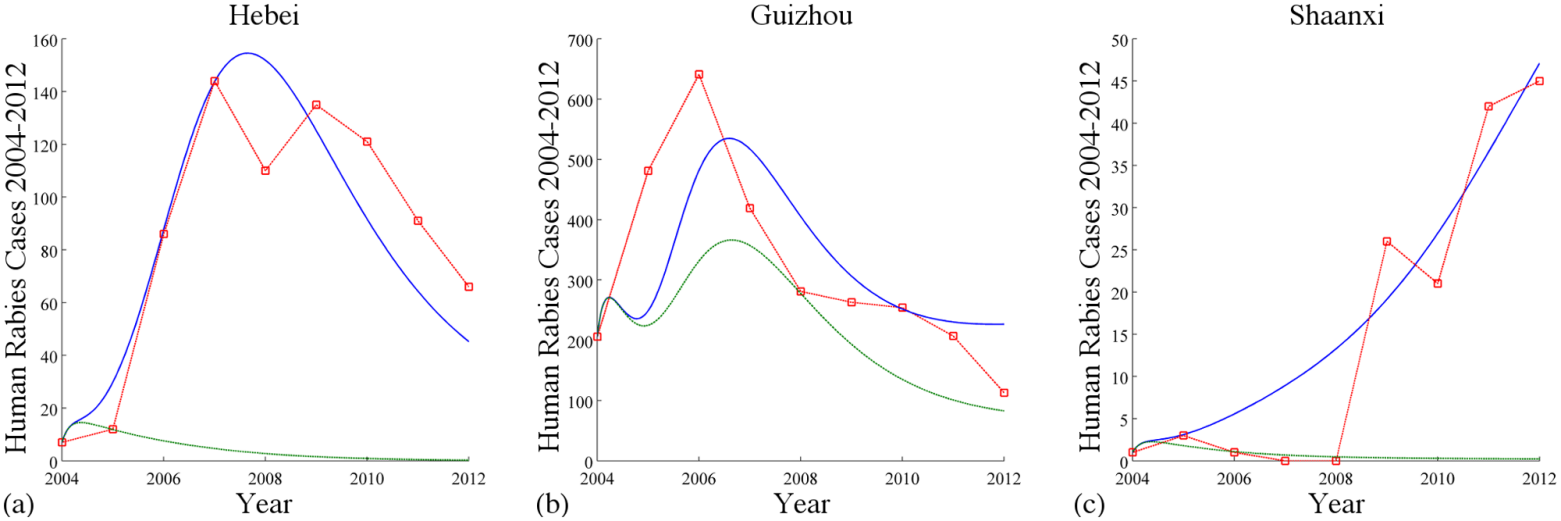
Simulations of the numbers of reported human rabies cases in Guizhou, Hebei, and Shaanxi with dog immigration and without dog immigration. The dashed red curves are reported cases by China CDC; the solid blue curves correspond to simulations with immigration and the dashed green curves correspond to simulations without immigration ($\phi_{12}^{K}=\phi_{21}^{K}=0$ for *K* = *S*, *E*, *I*, *V*).

Additionally, we show some direct comparisons of numerical simulations on the number of human cases from the model with immigration and without immigration. The additional green curves represent simulations of the human cases without any immigration in Hebei, Guizhou and Shaanxi, respectively. In Hebei, Fig 8(a) indicates the human infectious population size goes to zero faster without immigration which is consistent with the fact that the isolated basic reproduction number (0.5477) in Hebei is less than one. Similarly result can be observed in Fig 8(b) for Guizhou. Furthermore, Fig 8(c) shows that if there is no dog immigration in Shaanxi, the human rabies cases would decrease fast while it increased fast in reality.

### Sensitivity analysis

We now study how the basic reproduction number ℛ_0_ depends on parameters of dogs, especially the immigration rates $\phi_{ij}^{K}$, where *K* = *S*, *E*, *I*, *V*. For the sake of implicity, we consider the two-patch submodel and the corresponding basic reproduction number given in Eq (3). We consider the following three cases.

**Fig 9:**
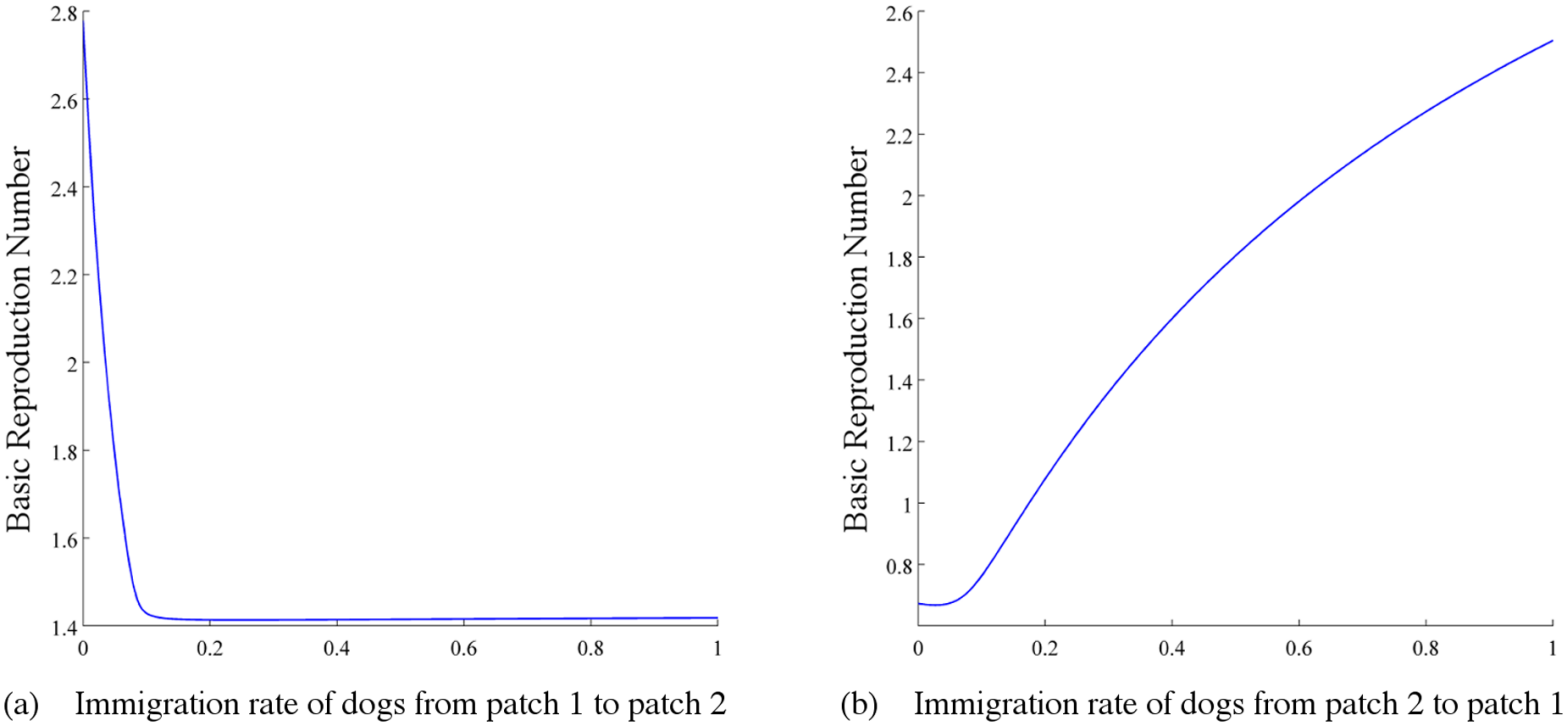
Plots of ℛ_0_ in terms of (a) the immigration rate of dogs from patch 1 to patch 2 (*ϕ*_21_) and (b) the immigration rate of dogs from patch 2 to patch 1 (*ϕ*_12_) when patch 1 has a higher transmission coefficient than patch 2 ($\beta_{1}^{D}=3\times 10^{7}>\beta_{2}^{D}=1\times 10^{7}$). Values of other parameters: *A*_1_ = *A*_2_ = 2 × 10^6^, $\lambda_{1}^{D}=\lambda_{2}^{D}=0.42$, $\sigma_{1}^{D}=\sigma_{2}^{D}=0.42$, $\gamma_{1}^{D}=\gamma_{2}^{D}=0.4$, $m_{1}^{D}=m_{2}^{D}=0.08$, $k_{1}^{D}=k_{2}^{D}=0.09$, $\mu_{1}^{D}=\mu_{2}^{D}=1$.

*(i) Immigration of dogs between patches with different transmission rates.* Suppose $\beta_{1}^{D}=3\times 10^{-7}>\beta_{2}^{D}=1\times 10^{-7}$, $\phi_{12}^{K}=\phi_{12}$ and $\phi_{21}^{K}=\phi_{21}$, where *K* = *S*, *E*, *I*, *R*. *A*_1_ = 2 × 6^6^, $\lambda_{1}^{D}=0.42$, $\sigma_{1}^{D}=0.42$, $\gamma_{1}^{D}=0.4$, $m_{1}^{D}=0.08$, $k_{1}^{D}=0.09$, $\mu_{1}^{D}=1$, the remaining parameters of dogs in patch 2 are the same as the corresponding parameters of dogs in patch 1. Here the only difference between the two patches in that the transmission coefficients of infectious dogs to susceptible dogs are different. Then the isolated basic production numbers satisfy the inequality: ${ℛ}_{0}^{1}=2.3246>{ℛ}_{0}^{2}=0.7749$. So rabies is endemic in patch 1 and will die out in patch 2. First, let (the immigration rate of dogs from patch 1 to patch 2) *ϕ*_12_ = 0.02. It is shown in Fig 9 that ℛ_0_ decreases as *ϕ*_21_ (the immigration rate of dogs from patch 2 to patch 1) increases. Then, let *ϕ*_21_ = 0.5, ℛ_0_ increases as *ϕ*_12_ increases. Furthermore, if *ϕ*_21_ is small and *ϕ*_12_ is large, ℛ_0_ is greater than both ${ℛ}_{0}^{1}$ and ${ℛ}_{0}^{2}$. To reduce ℛ_0_, we need to control *ϕ*_12_ small enough. For example, let *ϕ*_21_ = 0.5, *ϕ*_12_ = 0.01, then we obtain that ${ℛ}_{0}<\min\{{ℛ}_{0}^{1},{ℛ}_{0}^{2}\}$. If *ϕ*_21_ = 0.4, *ϕ*_12_ = 0.3, then ℛ_0_ = 1.6274, which is smaller than ${ℛ}_{0}^{1}$ but greater than ${ℛ}_{0}^{2}$. Thus, if we can control the immigration rates of dogs in an appropriate range, the endemic level will be lower.

**Fig 10:**
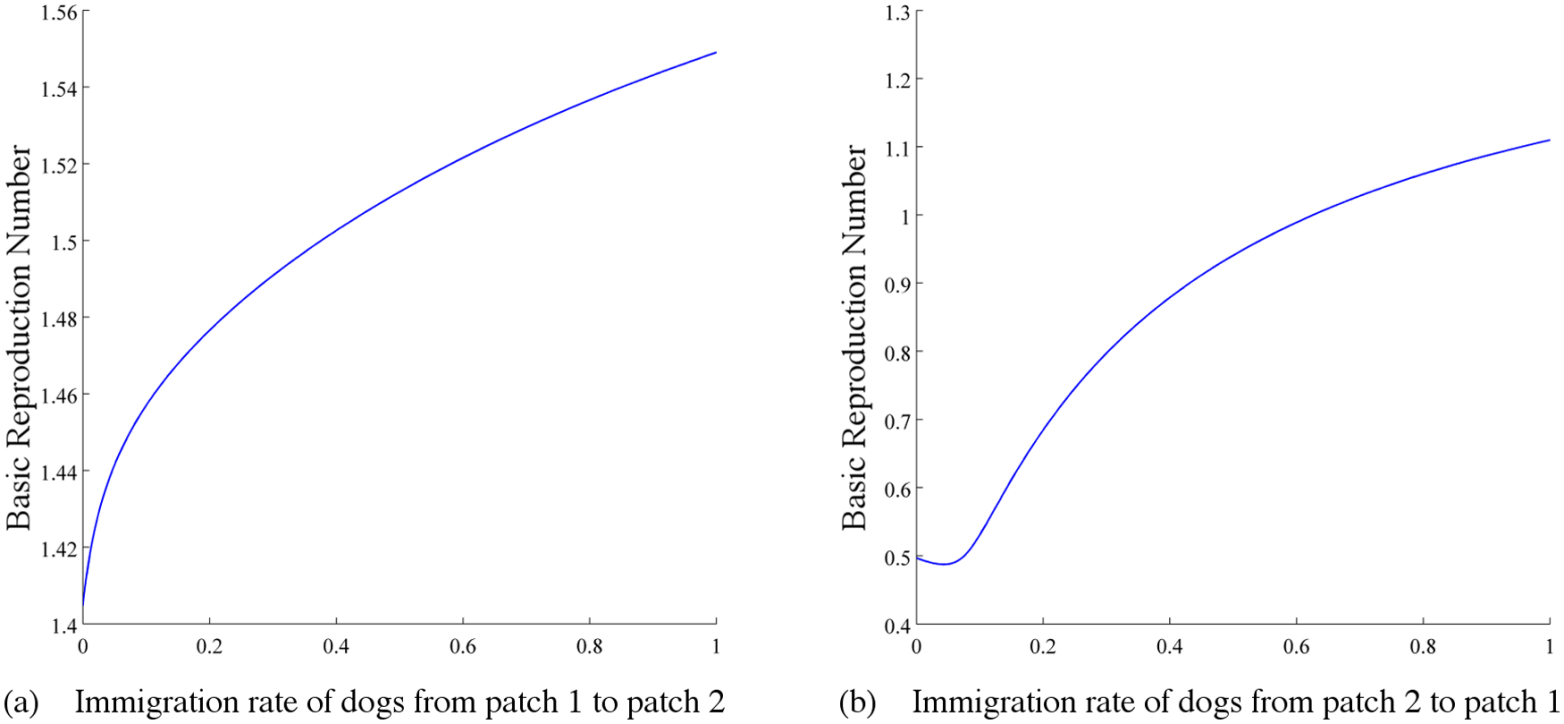
Plots of ℛ_0_ in terms of (a) the immigration rate of dogs from patch 1 to patch 2 (*ϕ*_21_) and (b) the immigration rate of dogs from patch 2 to patch 1(*ϕ*_12_) when patch 1 has a higher vaccination rate than patch 2 ($k_{1}^{D}=0.5>k_{2}^{D}=0.09$). We assume immigration rates of susceptible, exposed, infectious and vaccinated dogs are same, that is, $\phi_{21}=\phi_{21}^{S}=\phi_{21}^{E}=\phi_{21}^{I}=\phi_{21}^{V}$ and $\phi_{12}=\phi_{12}^{S}=\phi_{12}^{E}=\phi_{12}^{I}=\phi_{12}^{V}$. Values of other parameters: $\beta_{1}^{D}=\beta_{2}^{D}=1.58\times 10^{-7}$, *A*_1_ = *A*_2_ = 2 × 10^6^, $\lambda_{1}^{D}=\lambda_{2}^{D}=0.42$, $\sigma_{1}^{D}=\sigma_{2}^{D}=0.42$, $\gamma_{1}^{D}=\gamma_{2}^{D}=0.4$, $m_{1}^{D}=m_{2}^{D}=0.08$, $\mu_{1}^{D}=\mu_{2}^{D}=1$.

*(ii) Immigration of dogs between patches with different vaccination rates.* We assume that dogs move at the same rate regardless of their subclasses ($\phi_{12}^{K}=\phi_{12}$ and $\phi_{21}^{K}=\phi_{21}$ for *K* = *S*, *E*, *I*, *V*). Then let dogs in patch 1 have a higher vaccination rate than those in patch 2: $k_{1}^{D}=0.5>k_{2}^{D}=0.09$. All the remaining parameters of dogs in patch 2 are the same as the corresponding parameters of dogs in patch 1. Fig 10 presents the basic reproduction number ℛ_0_ in terms of the immigration rates. Firstly, ℛ_0_ increases as the immigration rates increase at most of the time. This is consistent with our previous simulation results: the dog movements bring difficulties to rabies control. Secondly, a detailed observation in the range of ℛ_0_ indicates that it is more sensitive in *ϕ*_12_. Therefore we conclude that immigration of dogs from the patch with lower vaccination rate to a patch with higher vaccination rate is more dangerous.

It is notable that ℛ_0_ might be greater than both isolated basic reproduction numbers. For example, let *ϕ*_21_ = 0.95 and *ϕ*_12_ = 0.4, and all other parameters be the same as in Case (ii). Then ${ℛ}_{0}=1.2974>\max\{{ℛ}_{0}^{1},{ℛ}_{0}^{2}\}$. That is, the immigration of dogs might lead to a more serious situation.

**Fig 11:**
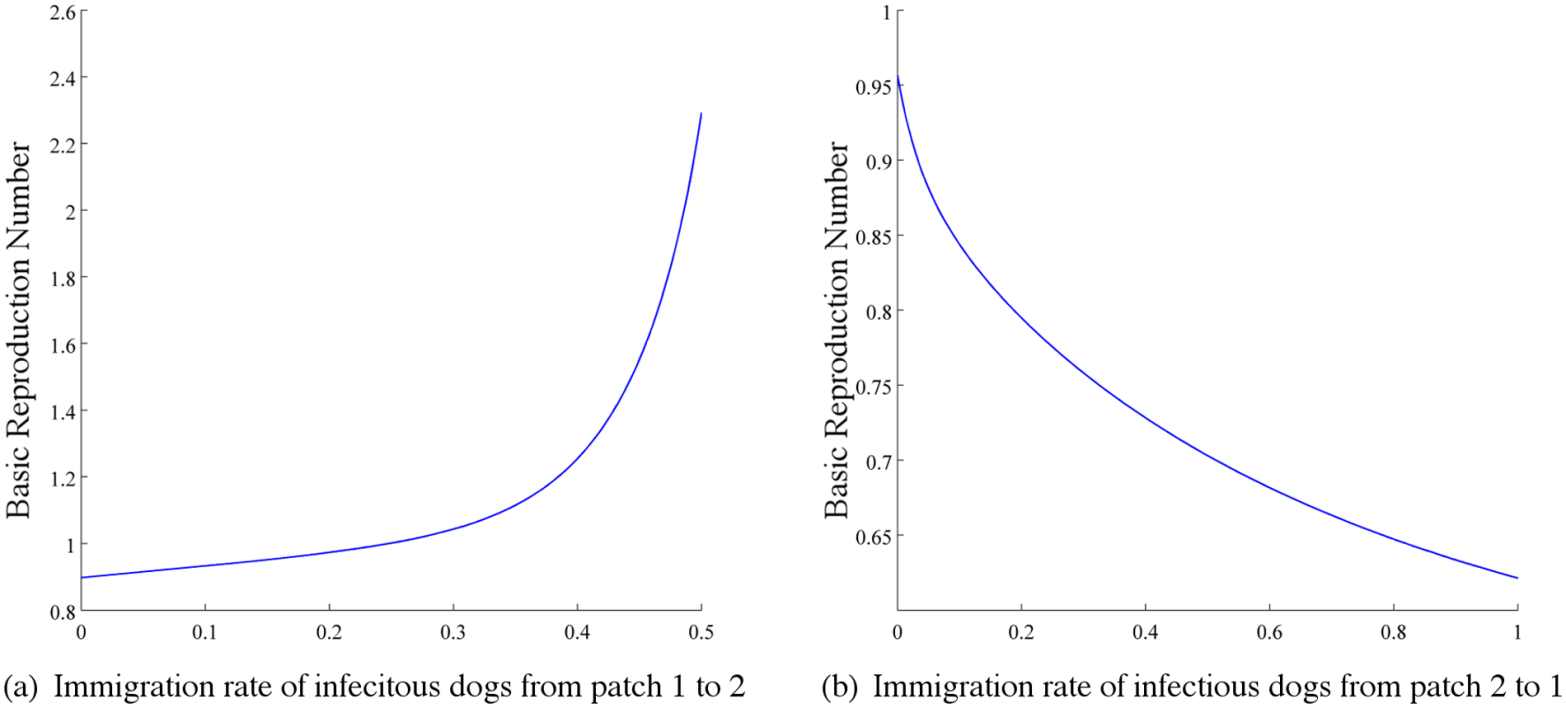
Plots of ℛ_0_ in terms of (a) immigration rate of infectious dogs from patch 1 to patch 2 ($\phi_{21}^{I}$) and (b) the immigration rate of infectious dogs from patch 2 to patch 1 ($\phi_{12}^{I}$) when patch 1 has a higher vaccination rate than patch 2 ($k_{1}^{D}=0.5>k_{2}^{D}=0.09$). Fix the immigration rates of susceptible, exposed and vaccinated dogs, that is, $\phi_{12}^{S}=\phi_{12}^{E}=\phi_{12}^{V}=0.2$, $\phi_{21}^{S}=\phi_{21}^{E}=\phi_{21}^{V}=0.2$. Values of other parameters: $\beta_{1}^{D}=\beta_{2}^{D}=1.58\times 10^{-7}$, *A*_1_ = *A*_2_ = 2 × 10^6^, $\lambda_{1}^{D}=\lambda_{2}^{D}=0.42$, $\sigma_{1}^{D}=\sigma_{2}^{D}=0.42$, $\gamma_{1}^{D}=\gamma_{2}^{D}=0.4$, $m_{1}^{D}=m_{2}^{D}=0.08$, $\mu_{1}^{D}=\mu_{2}^{D}=1$.

*(iii) Immigration of infective dogs between patches.* Now we fix all immigration rates of dogs to 0.2 except $\phi_{21}^{I}$ (the immigration rate of infective dogs from patch 1 to patch 2), then ℛ_0_ increases quickly as $\phi_{21}^{I}$ increases, as it is shown in Fig 11(a). On the other hand we fix all immigration rates of dogs to 0.2 except $\phi_{12}^{I}$ (the immigration rate of infective dogs from patch 2 to patch 1), then ℛ_0_ decreases as $\phi_{21}^{I}$ increases, as it is shown in Fig 11(b). Interestingly, compare with *Case ii*, we found that immigration of infectious dogs from the patch with a high vaccination rate to a patch with a low vaccination rate is more dangerous. The patch with a low vaccination rate actually has a week protection from the virus, thus infectious dogs from another patch may spread the disease faster.

## Discussion

In 1999, human rabies cases were reported in about 120 counties in mainland China, mainly in the southern provinces. Now outbreaks of human rabies have been reported in about 1000 counties and the disease has spread geographically from the south to the north. Phylogeographic analyses for rabies virus strains ([12, 14]) indicate that prevalent strains in northern provinces are indeed related to the remote southern provinces. It is believed that the geographical spread of rabies virus are caused by the transportation of dogs.

In this paper, a multi-patch model is proposed to describe the spatial transmission dynamics of rabies in China and to investigate how the immigration of dogs affects the geographical spread of rabies. The expression and sensitivity analysis of the basic reproduction number indicates that the movement of dogs plays an essential role in the spatial transmission of rabies. As mentioned in [8], reducing dog birth rate and increasing dog immunization coverage rate are the most effective methods in controlling human rabies infections in China. They also play important roles in controlling the spatial spread of rabies based on the multi-patch model. WHO (World Health Organization) recommends that 70% of dogs in a population should be immunized to eliminate the rabies. Unfortunately, this rate is still lower than 10% in most regions in China. Therefore, efforts to bring the awareness of the importance of treatments and enhance the vaccination coverage in dogs are important to control the disease in China.

We also performed some numerical simulations to study the effects of the immigration rate in three pairs of provinces in China: Guizhou and Guangxi, Hebei and Fujian, Sichuan and Shaanxi, as shown in Fig 2. First of all, the immigration may lead a basic reproduction number to be larger than one even if the isolated basic reproduction numbers are all less than one. Therefore, the immigration of dogs is the main factor for the long-distance inter-provincial spread of rabies. We note that the transportation of dogs even between non-endemic provinces, such as Fujian and Hebei, can cause human rabies in Hebei to increase greatly. Additionally, the movement of dogs from regions with a low vaccination rate also makes the situation worse. Attention should be paid not only to the provinces with more reported cases but also to the provinces with low vaccination rates. In those extremely poor areas, where dogs have a low vaccination coverage, the dog trade business and transportation to other areas will contribute to the geographical spread of rabies significantly. To control the disease at a national level, more efforts are needed in these regions.

The primary purpose of the transportation of dogs in China is believed to be related to food business. In some areas, such as the endemic provinces Guizhou and Guangxi, people eat dogs due to minority culture or harsh climate. There is no open market for selling and buying dogs for business purpose, however the black market always exists. It is frequently reported that trucks sometimes full of dogs are intercepted by animal lovers in the inter-provincial highway. Sometimes more than one thousand dogs were crammed into many tiny cages in one truck. The efficiency of such dog transportation has been enhanced by the fast development and expansion of the highway system in China in the past ten years. Chinese law requires that the transported animals must be certified as vaccinated for rabies and other diseases. However, dog traders are found to falsify the paperwork for most of the dogs in the truck to reduce their cost. Thus it would be important to regulate the market and implement certain policies on dogs (such as vaccine records) and the dog traders (such as licenses). During our research, we found that it was very difficult to find the information on dog population in China due to the lack of dog registration management. Since a large number of dogs are transported from provinces to provinces, it is necessary to register and manage such transportation properly. In particular, dogs carrying rabies viruses can easily spread the virus to other dogs when they are crowded into a small space during the trip. The last case of our sensitivity analysis shows the oblivious dangers resulted from the transportation of infectious dogs that has a destination with a low vaccination rate. We suggest creating strict and uniform procedures to test the dogs that will be transported.

We used a deterministic system to study the geographical spread of rabies in China and simulated the annual data in some provinces. Stochasticity is not considered in our model, and we also think seasonality plays an important role in the transmission of rabies. Therefore a mathematcal model which includes certain randomness and seasonality may help us to understand this problem better. Meanwhile, we only applied two-patch model to simulate the data in two provices. A more general case which can discuss the complex transmission among three or more provinces is interesting to study.

Chinese government has devoted a large amount of financial resource to the control of rabies, particularly in vaccinations. According to the statistics reported in “Chinese Rabies Prevention and Control Status” ([17]), about 12–15 million doses of human rabies vaccines are administered in China each year, accounting for 80 percent of the total global consumption. The production and administration of human rabies vaccines cost the country more than RMB 10 billion ($1.56 billion) each year. However, most of these efforts focused on humans and the vaccination rate of dogs in China still remains low. Under this high-risk environment for rabies, the only way to reduce deaths caused by rabies is to provide treatment immediately to exposures (contacts with category II and III). Then the total cost could be about RMB 24.5 billion annually if all of these exposures receive PEP treatments. Remarkably, the vaccines for dogs are less expensive than that for humans, but the dog vaccination implementation requires a continuously huge human, material and financial resources. It will be interesting to investigate how to optimize the resources and efforts and how to take the socioeconomic factors into consideration in order to pursue the control and elimination of rabies virus in humans.

## Supporting Information

S1 Text Mathematical model, disease-free equilibruim, and basic reproduction number. (PDF)
