# Supplementary material for: Modeling the Geographic Spread of Rabies in China
Source: PLoS Negl Trop Dis. 2015 May 28;9(5):e0003772. doi: 10.1371/journal.pntd.0003772 (PMC4447479; doi:10.1371/journal.pntd.0003772)
Supplement: S1 Text — (PDF) [file pntd.0003772.s001.pdf]

Supporting Text

Modelling the Geographic Spread of Rabies in China

Jing Chen[1], Lan Zou[2], Zhen Jin[3], Shigui Ruan[1,*],

1 Department of Mathematics, University of Miami, Coral Gables, FL 33124, USA

2 Department of Mathematics, Sichuan University, Chengdu, Sichuan 610064, P. R. China

3 Complex Systems Research Center, Shanxi University, Taiyuan, Shaanxi 03006, P. R. China

*E-mail: ruan@math.miami.edu

Mathematical model

The variables are listed as follows:

$S_i^D(t)$ – the population of susceptible dogs at time t in patch i;

$E_i^D(t)$ – the population of exposed dogs at time t in patch i;

$I_i^D(t)$ – the population of infectious dogs at time t in patch i ;

$V_i^D(t)$ – the population of vaccinated dogs at time t in patch i;

$S_i^H(t)$ – the population of susceptible humans at time t in patch i;

$E_i^H(t)$ – the population of exposed humans at time t in patch i;

$I_i^H(t)$ – the population of infectious humans at time t in patch i ;

$V_i^H(t)$ – the population of vaccinated humans at time t in patch i.

For the sake of convenience, we re-state the model as follows:

$$
\begin{aligned}
\frac{dS_i^D}{dt} &= A_i + \lambda_i^D V_i^D + \sigma_i^D(1-\gamma_i^D)E_i^D - \beta_i^D S_i^D I_i^D - (m_i^D + k_i^D)S_i^D + \sum_{j=1}^{n} \phi_{ij}^S S_j^D,\\
\frac{dE_i^D}{dt} &= \beta_i^D S_i^D I_i^D - (m_i^D + \sigma_i^D + k_i^D)E_i^D + \sum_{j=1}^{n} \phi_{ij}^E E_j^D,\\
\frac{dI_i^D}{dt} &= \sigma_i^D \gamma_i^D E_i^D - (m_i^D + \mu_i^D)I_i^D + \sum_{j=1}^{n} \phi_{ij}^I I_j^D,\\
\frac{dV_i^D}{dt} &= k_i^D(S_i^D + E_i^D) - (m_i^D + \lambda_i^D)V_i^D + \sum_{j=1}^{n} \phi_{ij}^V V_j^D,\\
\frac{dS_i^H}{dt} &= B_i + \lambda_i^H V_i^H + \sigma_i^H(1-\gamma_i^H)E_i^H - m_i^H S_i^H - \beta_i^H S_i^H I_i^D + \sum_{j=1}^{n} \psi_{ij}^S S_j^H,\\
\frac{dE_i^H}{dt} &= \beta_i^H S_i^H I_i^D - (m_i^H + \sigma_i^H + k_i^H)E_i^H + \sum_{j=1}^{n} \psi_{ij}^E E_j^H,\\
\frac{dI_i^H}{dt} &= \sigma_i^H \gamma_i^H E_i^H - (m_i^H + \mu_i^H)I_i^H + \sum_{j=1}^{n} \psi_{ij}^I I_j^H,\\
\frac{dV_i^H}{dt} &= k_i^H E_i^H - (m_i^H + \lambda_i^H)V_i^H + \sum_{j=1}^{n} \psi_{ij}^V V_j^H.
\end{aligned}
\tag{1}
$$

Ignore the deaths and births during transportation. For $i = 1, ..., n$, we have

$$
\phi_{ii}^K = -\sum_{j=1, j\neq i}^{n} \phi_{ji}^K, \quad K = S, E, I, V, \quad \psi_{ii}^L = -\sum_{j=1, j\neq i}^{n} \psi_{ji}^L, \quad L = S, E, I, V.
$$

Assume that the travel rate matrices $(\phi_{ij}^K)_{n\times n}$ for $K = S, E, I, V$ and $(\psi_{ij}^L)_{n\times n}$ for $L = S, E, I, V$ are irreducible.

Desease-free equilibruim

Consider the case that $E_i^D = I_i^D = E_i^H = I_i^H = 0$, then the positive equilibria of the subsystem satisfy the following:

$$\begin{aligned} &A_i + \lambda_i^D V_i^D - (m_i^D + k_i^D) S_i^D + \textstyle\sum_{j=1}^n \phi_{ij}^S S_j^D = 0, \\ &k_i^D S_i^D - (m_i^D + \lambda_i^D) V_i^D + \textstyle\sum_{j=1}^n \phi_{ij}^V V_j^D = 0, \end{aligned} \tag{2}$$

and

$$\begin{aligned} &B_i + \lambda_i^H V_i^H - m_i^H S_i^H + \textstyle\sum_{j=1}^n \psi_{ij}^S S_j^H = 0, \\ &-(m_i^H + \lambda_i^H) V_i^H + \textstyle\sum_{j=1}^n \psi_{ij}^V V_j^H = 0. \end{aligned} \tag{3}$$

By adding the equations in (0.3) from $i = 1$ to n, we have

$$0 = \sum_{i=1}^n -(m_i^H + \lambda_i^H) V_i^H + \sum_{i=1}^n \sum_{j=1}^n \psi_{ij}^S S_j^H = -\sum_{i=1}^n (m_i^H + \lambda_i^H) V_i^H,$$

which leads to $V_i^H = 0$ for $i = 1, 2, ..., n$ and $B_i - m_i^H S_i^H + \sum_{j=1}^n \psi_{ij}^S S_j^H = 0$. It follows that there exists $S^{H*} = (S_1^{H*}, S_2^{H*}, ..., S_n^{H*})$ such that $(S^{H*})^T = (\delta_{ij} m_i^H - \psi_{ij}^S)^{-1} (B_1, B_2, ..., B_n)^T$ since the matrix $(\delta_{ij} m_i^H - \psi_{ij}^S)$ is strictly diagonal dominant.

Let $X = (S_1^D, S_2^D, ..., S_n^D, V_1^D, V_2^D, ..., V_n^D)^T$. Then equation (0.2) is equivalent to $A + MX = 0$, where $A = (A_1, A_2, ..., A_n, 0, 0, ..., 0)^T$ and

$$M = \begin{pmatrix} M_{11} & M_{12} \\ M_{21} & M_{22} \end{pmatrix},$$

here

$$\begin{aligned} M_{11} &= (-\delta_{ij}(m_i^D + k_i^D) + \phi_{ij}^S)_{n \times n}, \\ M_{12} &= (\delta_{ij} \lambda_i)_{n \times n} = \text{diag}(\lambda_1^D, \lambda_2^D, ..., \lambda_n^D), \\ M_{21} &= (\delta_{ij} k_i^D)_{n \times n} = \text{diag}(k_1^D, k_2^D, ..., k_n^D), \\ M_{22} &= (-\delta_{ij}(m_i^D + \lambda_i^D) + \phi_{ij}^V)_{n \times n}. \end{aligned}$$

Consider the i-th column of matrix M, we can show that

$$|-\delta_{ij}(m_i^D+k_i^D)+\phi_{ii}^S| = \delta_{ij}(m_i^D+k_i^D)+\sum_{j\neq i}\phi_{ji}^S > \delta_{ij}k_i^D+\sum_{j\neq i}\phi_{ji}^S.$$

After showing the similar properties for the i-th column where $i=n+1,n+2,...,2n$, we can see that M is strictly diagonal dominant. Therefore, it implies the existence of the inverse of M. We denote $(S^{D*},V^{D*})=-M^{-1}A$, where

$$S^{D*}=(S_1^{D*},S_2^{D*},...,S_n^{D*}),$$
$$V^{D*}=(V_1^{D*},V_2^{D*},...,V_n^{D*}).$$

Also, denote

$$S^{H*}=(S_1^{H*},S_2^{H*},...,S_n^{H*}).$$

We thus obtain the disease-free equilibrium for model (0.1)

$$(S^{D*},0,0,V^{D*},S^{H*},0,0,0).$$

Basic reproduction number

Following van den Driessche and Watmough [22], we obtain that

$$\mathcal{F}=\begin{pmatrix}0&\mathcal{F}_1&0&0\\0&0&0&0\\0&\mathcal{F}_2&0&0\\0&0&0&0\end{pmatrix},\quad \mathcal{V}=\begin{pmatrix}\mathcal{V}_{11}&0&0&0\\\mathcal{V}_{21}&\mathcal{V}_{22}&0&0\\0&0&\mathcal{V}_{33}&0\\0&0&\mathcal{V}_{34}&\mathcal{V}_{44}\end{pmatrix},$$

where

$$\mathcal{F}_1 = \text{diag}(\beta_1^D S_1^{D*}, \beta_2^D S_2^{D*}, ..., \beta_n^D S_n^{D*});$$

$$\mathcal{F}_2 = \text{diag}(\beta_1^H S_1^{H*}, \beta_2^H S_2^{H*}, ..., \beta_n^H S_n^{H*});$$

$$\mathcal{V}_{11} = (\delta_{ij}(m_i^D + \sigma_i^D + k_i^D) - \phi_{ij}^E)_{n\times n};$$

$$\mathcal{V}_{22} = (\delta_{ij}(m_i^D + \mu_i^D) - \phi_{ij}^I)_{n\times n};$$

$$\mathcal{V}_{33} = (\delta_{ij}(m_i^H + \sigma_i^D + k_i^H) - \psi_{ij}^E)_{n\times n};$$

$$\mathcal{V}_{44} = (\delta_{ij}(m_i^H + \mu_i^H) - \psi_{ij}^I)_{n\times n};$$

$$\mathcal{V}_{21} = \text{diag}(\sigma_1^D \gamma_1^D, \sigma_2^D \gamma_2^D, ..., \sigma_n^D \gamma_n^D);$$

$$\mathcal{V}_{21} = \text{diag}(\sigma_1^H \gamma_1^H, \sigma_2^H \gamma_2^H, ..., \sigma_n^H \gamma_n^H).$$

Since A_{ii} is a strictly diagonally dominant matrix for $i = 1, 2, ..., n$, they are all non-singular and therefore A_{ii}^{-1} exists. That leads to the inverse of V:

$$\mathcal{V}^{-1} = \begin{pmatrix} \mathcal{V}_{11}^{-1} & 0 & 0 & 0 \\ \mathcal{V}_{22}^{-1}\mathcal{V}_{21}\mathcal{V}_{11}^{-1} & \mathcal{V}_{22}^{-1} & 0 & 0 \\ 0 & 0 & \mathcal{V}_{33}^{-1} & 0 \\ 0 & 0 & \mathcal{V}_{44}^{-1}\mathcal{V}_{34}\mathcal{V}_{33}^{-1} & \mathcal{V}_{44}^{-1} \end{pmatrix}.$$

Thus, the spectral radius of matrix $\mathcal{F}\mathcal{V}^{-1}$,

$$\mathcal{R}_0 = \rho(\mathcal{F}\mathcal{V}^{-1}) = \rho(\mathcal{F}_1\mathcal{V}_{22}^{-1}\mathcal{V}_{21}\mathcal{V}_{11}^{-1}), \tag{4}$$

is the basic reproduction number.
